# Ferroptosis-related lncRNAs signature to predict the survival and immune evasion for lung squamous cell carcinoma

**DOI:** 10.3389/fgene.2022.968601

**Published:** 2022-08-26

**Authors:** Rusi Zhang, Xuewen Zhang, Han Yang, Yongbin Lin, Yingsheng Wen, Dechang Zhao, Lianjuan Chen, Peng Lin, Lanjun Zhang

**Affiliations:** ^1^ State Key Laboratory of Oncology in South China, Collaborative Innovation Center for Cancer Medicine, Guangzhou, China; ^2^ Department of Thoracic Surgery, Sun Yat-sen University Cancer Center, Guangzhou, China; ^3^ Department of Anesthesiology, Sun Yat-sen University Cancer Center, Guangzhou, China

**Keywords:** lung cancer, ferroptosis, lncRNA, tumor immune microenvironment, T cell dysfunction, immune evasion

## Abstract

**Introduction:** the investigation on the interactions between ferroptosis and lncRNAs for lung squamous cell carcinoma (LUSC) has been scare, and its impact on tumor immune microenvironment remained unknown. We aim to not only identify a ferroptosis-related lncRNAs signature for LUSC prognosis, but also evaluate its correlation to tumor immune evasion.

**Methods:** RNA sequencing data and survival information were obtained from The Cancer Genome Atlas database. A ferroptosis-related lncRNAs signature (FerRLSig) was developed and validated by univariate Cox regression, Least Absolute Shrinkage and Selection Operator regression and multivariate Cox regression. The tumor immune microenvironment and immune evasion were subsequently evaluated based on the FerRLSig stratification.

**Results:** the FerRLSig consisted of 10 ferroptosis-related lncRNAs and significantly associated with overall survival with satisfactory area under curve (HR = 2.240, 95% CI: 1.845–2.720, *p* < 0.001, 5-years AUC: 0.756). Based on the FerRLSig stratification, the high-risk group demonstrated not only significantly higher immune infiltration, but also more profound T cell dysfunction and immune evasion, which might ultimately lead to the resistance to current immune checkpoint inhibitors.

**Conclusion:** a robust prognostic FerRLSig for LUSC has been developed and validated, demonstrating a close association not only with tumor immune cell infiltration, but also with T cell dysfunction and immune evasion. Further investigation is warranted to better improve the survival of LUSC patients based on the FerRLSig stratification.

## Introduction

Lung cancer has been the top one cause of cancer death with one of the highest incidence rates second only to breast cancer worldwide (Sung et al., 2021). Lung squamous cell carcinoma (LUSC) accounts for approximately 20% of all lung cancer cases and constitutes the bulk of non-small cell lung cancer with lung adenocarcinoma (Barta et al., 2019). There has been numerous effective targeted therapies for lung adenocarcinoma, which has significantly prolonged the survival of lung adenocarcinoma patients with certain mutations (Sordella et al., 2004; Solomon et al., 2014; Ramalingam et al., 2020; Shaw et al., 2020; Wu et al., 2020). Moreover, a large number of researches have utilized the transcriptome data of lung adenocarcinoma to build various prognostic and predictive tools, to derive useful risk stratification, and to provide valuable insights on the development of sensitive drugs (Guo et al., 2021; Lu et al., 2021; Zheng et al., 2021). However, compared to lung adenocarcinoma, LUSC lacks effective targeted therapy and is generally less well-defined in terms of gene expression profile.

On the other hand, tumor immune evasion has been identified as one of the hallmarks of cancer and closely related to the tumor immune microenvironment (Hanahan and Weinberg, 2011; Gajewski et al., 2013). And fortunately, immune checkpoint inhibitors tackling tumor immune evasion have made remarkable breakthroughs in LUSC and significantly improved the LUSC patients’ survival (Brahmer et al., 2015; Paz-Ares et al., 2018). But still some LUSC patients were resistant to the current treatments including immunotherapy, leading to intractable progression or relapse, and ultimately cancer death. Multiple studies have identified increased CD8^+^ T cell infiltration as a favorable prognostic factor (Lee and Ruppin, 2019; Morad et al., 2021). However, T cell dysfunction has also been recognized as an important mechanism of immunotherapy resistance (Thommen and Schumacher, 2018). Therefore, further evaluation of the LUSC gene expression pattern’s impact on tumor immune microenvironment and immune evasion are still needed for LUSC patients.

Ferroptosis is an iron-dependent, oxidatively regulated cell death activated by extrinsic blockade of the cystine/glutamate transporter or intrinsic blockade of intracellular antioxidants. Recent studies have demonstrated that ferroptosis plays a significant part in tumorigenesis and various treatment sensitivity, thus might be a useful tool in cancer prognosis and patient stratification (Dixon et al., 2012; Chen et al., 2021). Moreover, long non-coding RNA (lncRNA), with more than 200 nucleotide and without functional protein translation, has been found to be closely related to tumorigenesis and tumor progression *via* ferroptosis in recent studies (Mao et al., 2018; Wang et al., 2019; Zhang et al., 2020; Statello et al., 2021). Therefore, interactions between ferroptosis and lncRNAs are likely to be critical to overcome cancer progression. However, investigation on the ferroptosis-related lncRNAs signature on LUSC has been scare and its impact on tumor immune microenvironment remained unknown, thus warranting further investigation.

In this study, we utilized The Cancer Genome Atlas database on lung squamous cell carcinoma (TCGA-LUSC) (Cancer Genome Atlas Research Network, 2012), and we aimed to develop a ferroptosis-related lncRNAs signature for LUSC prognosis. Furthermore, explorations on tumor immune microenvironment, tumor immune evasion and T cell dysfunction were also performed based on the FerRLSig stratification.

## Materials and methods

### Data acquisition

RNA sequencing data of TCGA-LUSC patients and all available clinical data were downloaded from the Genomic Data Commons portal (https://portal.gdc.cancer.gov/) (Grossman et al., 2016). Overall survival (OS) was calculated from the time of lung cancer diagnosis to the time of death or the last follow-up. Patients with incomplete survival information or with OS less than 30 days were excluded.

This is a retrospective study based on publicly available TCGA database. The Ethics Committee of our hospital has confirmed that no additional ethical approval or informed consent is required.

A list of 108 validated ferroptosis genes was obtained from FerrDb database (http://www.zhounan.org/ferrdb) (Zhou and FerrDb, 2020). LncRNAs was identified in TCGA-LUSC RNA sequencing data through GENCODE annotation (https://www.gencodegenes.org/) (Frankish et al., 2021). The correlations between 108 ferroptosis genes and the lncRNAs expression in the entire set of TCGA-LUSC were analyzed with Pearson’s correlation, and 4,259 ferroptosis-related lncRNAs were identified by the selection criterion of |Pearson R|) > 0.3 and *p* < 0.001 with any one of the 108 ferroptosis genes.

### Development and validation of the ferroptosis-related LncRNAs signature

The TCGA-LUSC patients were randomized into a training set and a testing set by the “caret” R package at the ratio of 7:3 (Kuhn, 2008). The FerRLSig was established with the training set while the validation was performed with the testing set and the entire set. Univariate Cox regression, Least Absolute Shrinkage and Selection Operator (LASSO) regression and multivariate Cox regression were applied in order to establish the final FerRLSig while avoiding overfitting with cross-validation (Friedman et al., 2010). The correlation between FerRLSig lncRNAs and ferroptosis genes was further visualized in both heatmap and Sankey diagram. The risk score of every patient was calculated as $\overset{n}{\underset{i=1}{\sum }}Coef\left(i\right)\times Expr\left(i\right)$ , with Coef(i) and Expr(i) representing the regression coefficient and expression level for each FerRLSig lncRNA respectively. The entire set of TCGA-LUSC patients were stratified into low-risk and high-risk groups by the median of the calculated risk scores.

### Further evaluation of the FerRLSig and establishment of the prognostic nomogram

The prognosis effect of the FerRLSig was further evaluated with Kaplan–Meier survival plot, subgroup analysis by universal clinical characteristics, correlation with universal clinical variables, univariate and multivariate Cox regression, and the area under the receiver operating characteristic curve (AUC). In addition, the discriminative ability of the FerRLSig was further evaluated through the comparison to the Principal component analysis (PCA) and the t-distributed stochastic neighbor embedding (t-SNE).

A nomogram incorporating the FerRLSig, age, gender and TNM stage, was developed to visualize the prognostic model and facilitate its clinical application for the OS of LUSC patients. The prognostic power of the nomogram was evaluated with AUC curves at 1, 3, and 5 years’ OS, calibration curves were also plotted and visually assessed. Furthermore, decision curve analysis (DCA) was also conducted to demonstrate the actual net benefit gain of the nomogram (Vickers and Elkin, 2006).

### Tumor immune microenvironment exploration and drug sensitivity screening based on the FerRLSig stratification

To analyze and compare the tumor immune microenvironment based on the FerRLSig stratification, xCell and ESTIMATE were both applied to inferred the immune and stromal cell infiltration (Yoshihara et al., 2013; Aran et al., 2017). In addition, to further characterize the potential underlying molecular pathways, gene set enrichment analysis (GSEA) was performed to identify the significantly enriched pathways in low-risk and high-risk groups respectively. The hallmark gene sets and C5 gene sets from the Gene Ontology (GO) were downloaded from the Molecular Signatures Database as the reference files (Subramanian et al., 2005). A nominal *p* value < 0.05 and a false discovery rate (FDR) q value < 0.25 were set as the statistically significant thresholds for GSEA GO analysis. Moreover, the expression level of several immune checkpoints and immune inhibitory factors, including CCL2, CD274 (PD-L1), CTLA4, CXCR4, IL6, LAG3, PDCD1 (PD-1), and TGFB1 were compared between low-risk and high-risk group. To further evaluate the predictive application for immune checkpoint inhibitors of the FerRLSig, TIDE score were compared between low-risk and high-risk groups (Fu et al., 2020). Furthermore, drug sensitivity screening was also performed with the 198 compounds available from the Genomics of Drug Sensitivity in Cancer (GDSC) database (Yang et al., 2013). And the half-maximal inhibitory concentration (IC50) of the available compounds on low-risk and high-risk groups was extracted with the Oncopredict R package (Maeser et al., 2021).

### Statistical analysis

Discrete variables were described as counts and percentages, their differences between groups were statistically evaluated with Pearson chi-square test or Fisher’s exact test (any expected values less than 5). On the other hand, continuous variables were described as median, mean and/or interquartile range, and their differences between groups were compared with Mann–Whitney–Wilcoxon test.

All statistical analyses and visualizations were performed with R version 4.1.0 (http://www.R-project.org) and corresponding packages. The Kaplan-Meier method was utilized in survival analysis and survival curves were compared with log-rank test. Two-sided *p* < 0.05 was considered statistically significant.

## Results

### Development and validation of the FerRLSig

**FIGURE 1:**
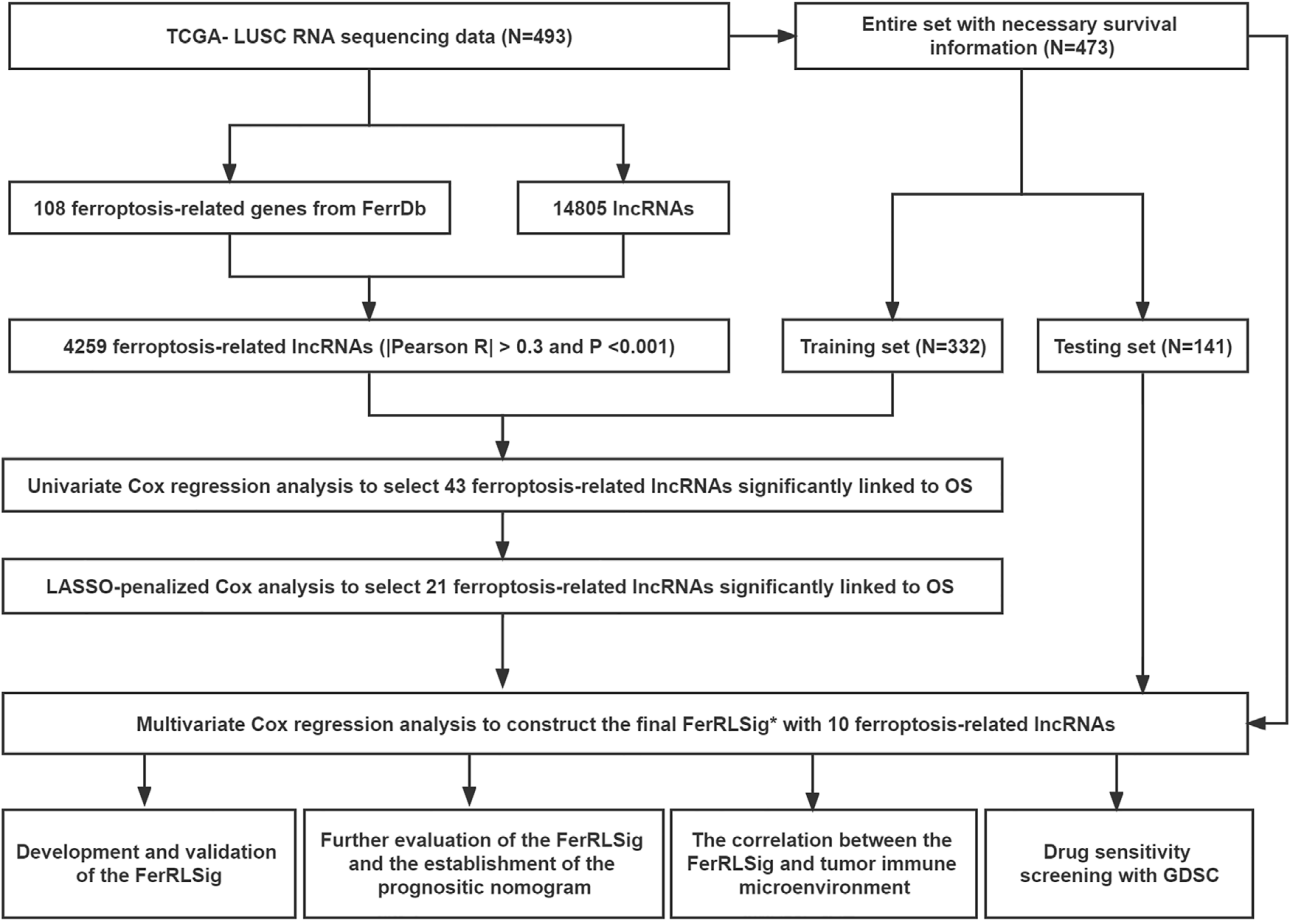
Flow chart of this study; *FerRLSig: the ferroptosis-related lncRNAs signature.

**FIGURE 2:**
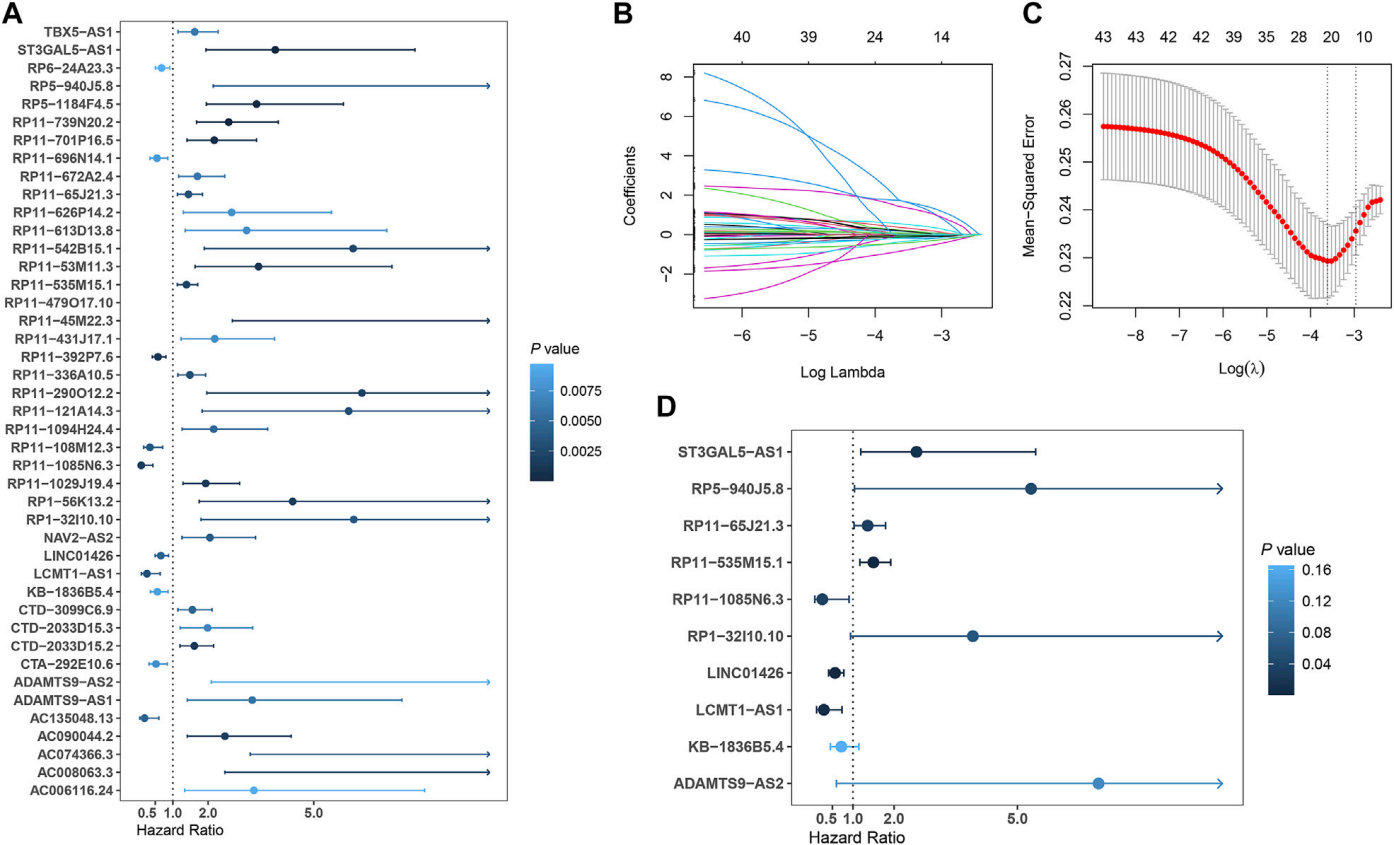
Development of prognostic ferroptosis-related lncRNAs signature for LUSC patients; **(A)** Forest plot of the 43 selected lncRNAs significantly associated with overall survival by univariate Cox regression analysis; **(B)** The coefficient profile of 21 OS-related lncRNAs chosen by LASSO regression; **(C)** The mean-squared error curve with different tuning parameters (logλ) and perpendicular dotted lines were drawn at the logλ corresponding to the minimum mean squared error (lambda.min) and the most regularized model within one standard error of the minimum mean squared error (lambda.1-se); **(D)** Forest plot of the 10 final selected lncRNAs by multivariate Cox regression analysis.

**FIGURE 3:**
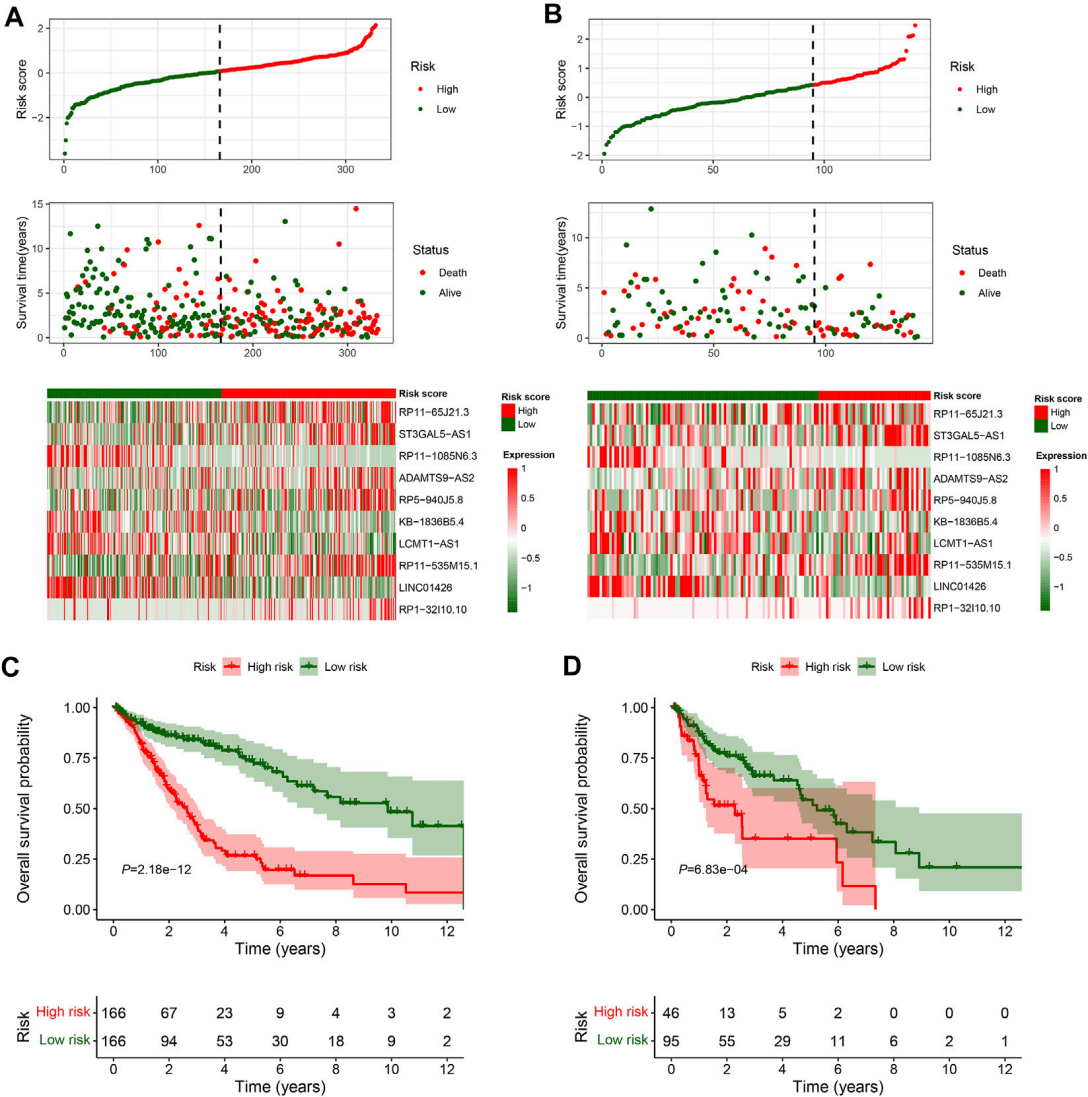
Overall survival analysis and validation of the ferroptosis-related lncRNAs signature; **(A–B)** Distribution of risk score, OS time, OS status and heatmap of the 10 prognostic ferroptosis-related lncRNAs signature in the TCGA-LUSC training set **(A)** and TCGA-LUSC testing set **(B)**. **(C–D)** Kaplan-Meier survival curves of the OS of the patients in the high- and low-risk groups for the TCGA-LUSC training set **(C)** and TCGA-LUSC testing set **(D)**.

In total, 493 patients with RNA sequencing data were downloaded from the TCGA-LUSC database, and 473 patients with necessary survival information were included and randomly assigned into training set and testing set by the ratio of 7:3 as demonstrated in the study workflow (Figure 1). The mean age of the patients in the entire set was 67 years old, 74.2% were male, 71.0% were Caucasian and 67.7% were early stage (I-II). No statistically significant difference was found between the training set and the testing set (Table 1). In the entire set, 4,259 lncRNAs were significantly correlated with any one of the 108 ferroptosis genes (|Pearson R|>0.3 and *p* < 0.001, Supplementary Figure S1A), among which 43 were significantly associated with OS by univariate Cox regression in the training set (*p* < 0.01, Figure 2A). Within the training set, 21 ferroptosis-related lncRNAs were further identified as significant prognostic factors *via* LASSO regression with the λ set at lambda. min that gave the minimum mean squared error (Figures 2B,C). The final FerRLSig was established with multivariate Cox regression, consisting of 10 lncRNAs (Figure 2D). The correlation between FerRLSig lncRNAs and ferroptosis genes were visualized in both heatmap and Sankey diagram for the entire set (Supplementary Figures S1B,C). The training set was divided into high-risk group and low-risk group by the median of the FerRLSig. Compared to low-risk group, high-risk group had evidently more death event and shorter overall survival time (Figure 3A). Within the FerRLSig, RP11-65J21.3, ST3GAL5-AS1, ADAMTS9-AS2, RP5-940J5.8, RP11-535M15.1, and RP1-32I10.10 were over-expressed in the high-risk group with positive coefficients and classified as risk promoters. On the other hand, RP11-1085N6.3, KB-1836B5.4, LCMT1-AS1, and LINC01426 were over-expressed in the low-risk group with negative coefficients and classified as risk inhibitors (Figure 3A; Supplementary Figure S1C). And the final FerRLSig Formula equal to 0.305*(RP11-65J21.3) + 0.934*(ST3GAL5-AS1) + (−1.366)*(RP11-1085N6.3) + 1.942*(ADAMTS9-AS2) + 1.674*(RP5-940J5.8) + (−0.331)*(KB-1836B5.4) + (−1.229)*(LCMT1-AS1) + 0.404*(RP11-535M15.1) + (−0.572)*(LINC01426) + 1.365*(RP1-32I10.10). The high-risk group was significantly associated with worse OS compared to low-risk group in the training set (HR = 3.345, *p* < 0.001, Figure 3C). For validation, the same model was applied to the testing set and the entire set, all FerRLSig lncRNAs demonstrated similar expression profiles in the high-risk and the low-risk groups. In addition, the high-risk group in both testing and entire set was also significantly associated with worse OS compared to low-risk group (testing set HR = 2.290, *p* < 0.001, Figures 3B,D; entire set HR = 2.606, *p* < 0.001, Supplementary Figure S2).

**TABLE 1:** The baseline characteristics of LUSC patients in TCGA database.

	Subgroups	Total, *n* = 473	Training set, *n* = 332	Testing set, *n* = 141	*p* Value
Age, mean(SD)		67 (9)	67 (8)	67 (9)	0.807
Gender, n (%)	Female	122 (25.8)	83 (25.0)	39 (27.7)	0.624
	Male	351 (74.2)	249 (75.0)	102 (72.3)	
Race, n (%)	Caucasian	336 (71.0)	228 (68.7)	108 (76.6)	0.104
	Other ethnicities	137 (29.0)	104 (31.3)	33 (23.4)	
Stage, n (%)	I-II	320 (67.7)	217 (65.4)	103 (73.0)	0.260
	III	74 (15.6)	56 (16.9)	18 (12.8)	
	IV	79 (16.7)	59 (17.8)	20 (14.2)	
T, n (%)	T1	108 (22.8)	71 (21.4)	37 (26.2)	0.587
	T2	276 (58.4)	200 (60.2)	76 (53.9)	
	T3	68 (14.4)	46 (13.9)	22 (15.6)	
	T4	21 (4.4)	15 (4.5)	6 (4.3)	
N, n (%)	N0	300 (63.4)	204 (61.4)	96 (68.1)	0.320*
	N1	125 (26.4)	89 (26.8)	36 (25.5)	
	N2	39 (8.2)	31 (9.3)	8 (5.7)	
	N3	9 (1.9)	8 (2.4)	1 (0.7)	
M, n (%)	M0	393 (83.1)	272 (81.9)	121 (85.8)	0.369
	M1	80 (16.9)	60 (18.1)	20 (14.2)	

*p value of fisher’s exact test for at least one expected count less than 5.

### Evaluation of the FerRLSig and the establishment of prognostic nomogram with the FerRLSig

**FIGURE 4:**
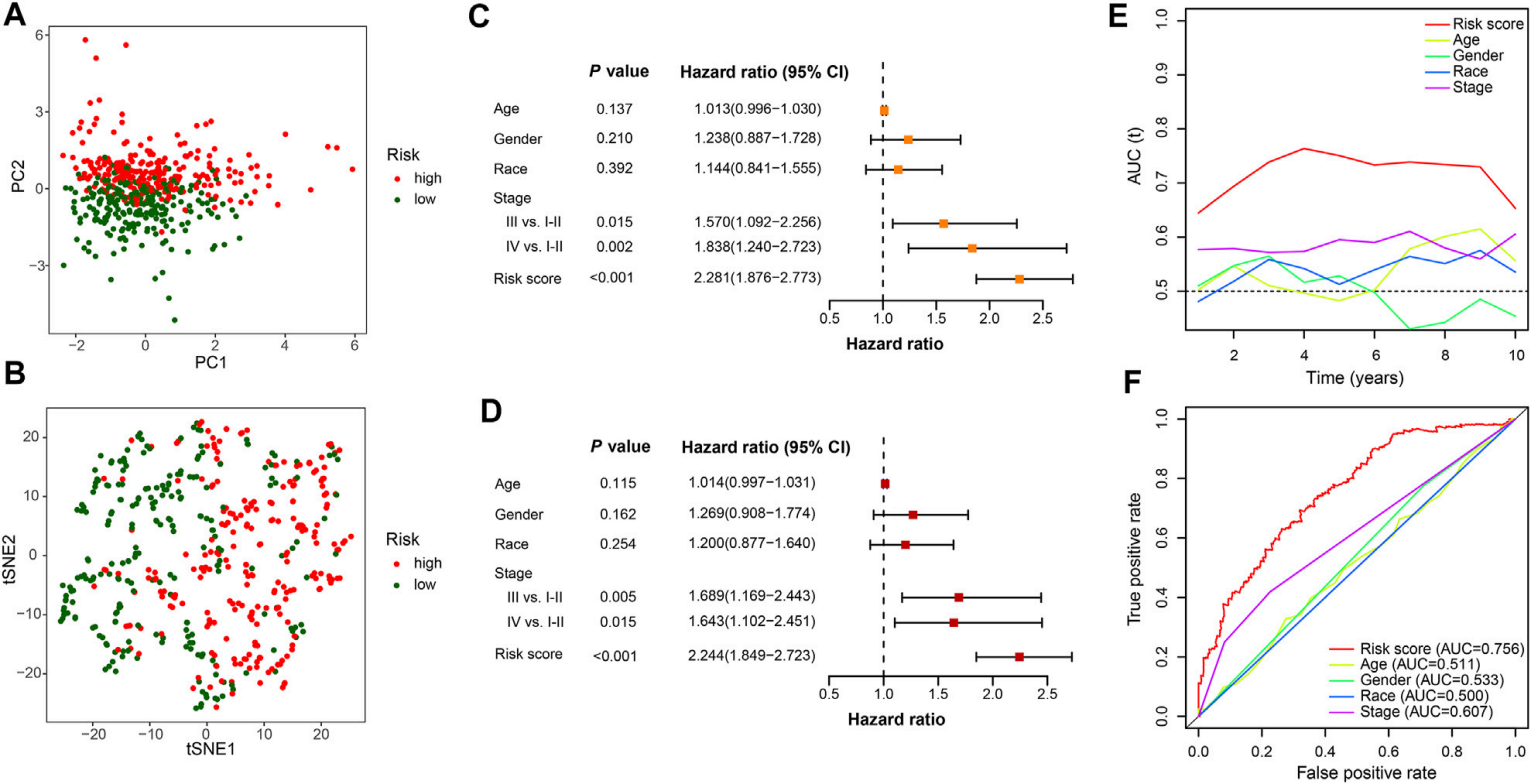
Evaluation of the ferroptosis-related lncRNAs signature (FerRLSig) in the entire set of TCGA-LUSC; **(A)** Principal component analysis of TCGA-LUSC RNA sequencing annotated with the FerRLSig stratification; **(B)** t-distributed stochastic neighbor embedding (t-SNE) analysis annotated with the FerRLSig stratification; **(C)** Univariate Cox regression overall survival analysis of the FerRLSig score and universal clinical characteristics; **(D)** Multivariate Cox regression overall survival analysis of the FerRLSig score and universal clinical characteristics; **(E)** Time-dependent area under curve plot of the risk score and clinical characteristics. **(F)** Receiver operating characteristic (ROC) curves of the universal clinical characteristics and risk score of the 5-years overall survival.

To compare the whole expression profile and the FerRLSig, both PCA and t-SNE were applied to the RNA sequencing of the entire set and annotated with the FerRLSig risk stratification. The high-risk group and low-risk group demonstrated distinctly different distribution in both PCA and t-SNE, indicating that the FerRLSig risk stratification recapitulated the major variability of the TCGA-LUSC RNA sequencing (Figures 4A,B). The correlations of the FerRLSig risk score with clinical characteristics including age, gender, and TNM stage were explored and no statistically significant correlation was found (Supplementary Figure S3). Univariate and multivariate Cox OS analysis were further performed with FerRLSig and other universal clinical characteristics. Both TNM stage and FerRLSig (univariate: HR = 2.281, *p* < 0.001; multivariate: HR = 2.240, *p* < 0.001) demonstrated significant prognostic effect in both univariate and multivariate Cox regression (Figures 4C,D). In addition, time-dependent AUC of the FerRLSig and other clinical characteristics were plotted, and the FerRLSig demonstrated consistently higher AUC compared to other clinical characteristics, including TNM stage (5-years AUC: FerRLSig 0.756, TNM stage 0.607, Figures 4E,F).

**FIGURE 5:**
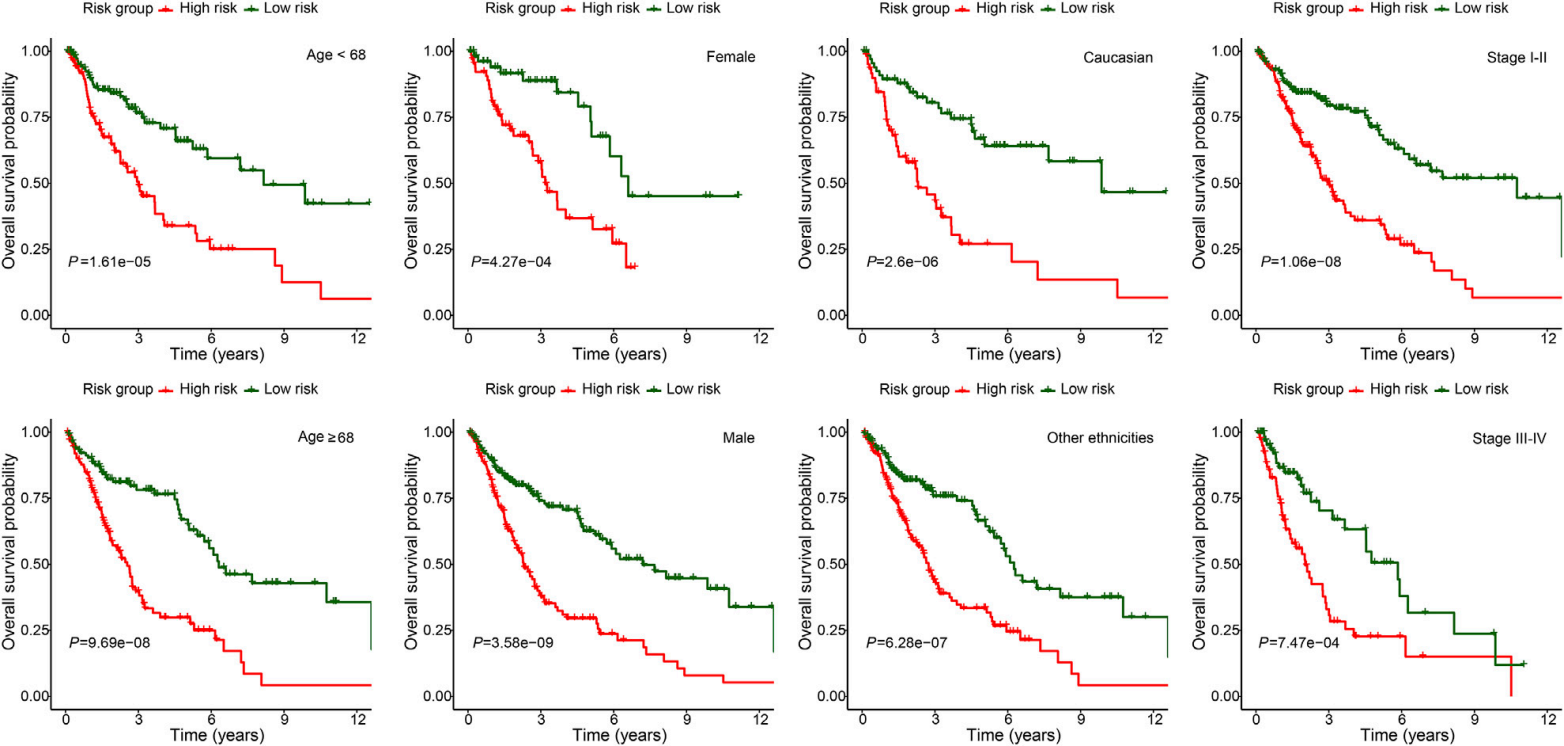
Subgroup overall survival analysis of the FerRLSig stratification by different age, gender, ethnicities and TNM stage subgroups in the TCGA-LUSC entire set.

To further demonstrate the model applicability in different population, subgroup OS analysis was performed in different age, gender, ethnicity and stage groups. The high-risk group was consistently associated with worse OS in all subgroups, which not only validated the model’s wide applicability in different population, but also demonstrated its independent prognostic role (Figure 5).

**FIGURE 6:**
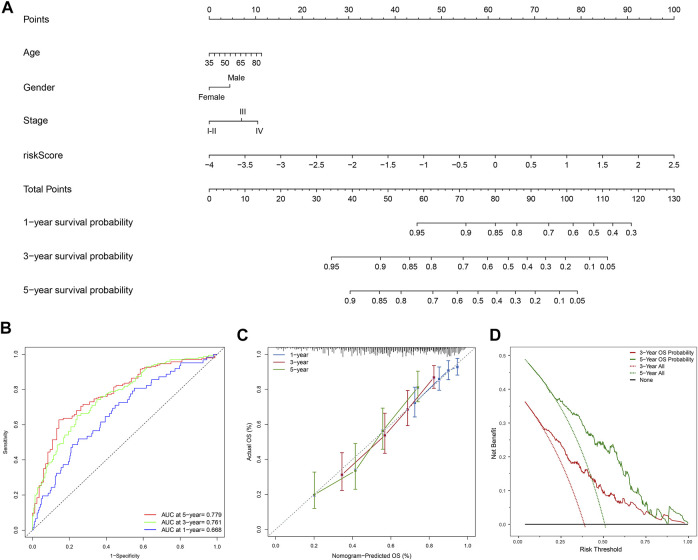
Development and evaluation of the prognostic nomogram; **(A)** A clinical prognostic nomogram was developed to predict the 1-, three- and 5-years overall survival (OS) probability. A vertical line between each variable and point scale can be drawn to determine the points for each variable, then all the points are summed up as the total points, and the predicted overall survival rate of the 1-, three- and 5-years were calculated by drawing a vertical line from the total points scale to the 1-, three- and 5-years survival scales; **(B)** Area under curve plot of the nomogram for the 1-, three- and 5-years OS; **(C)** Calibration curves of the nomogram for 1-, three- and 5-years overall survival: nomogram-predicted overall survival is plotted on the *x*-axis, actual overall survival is plotted on the *y*-axis, a plot along the 45-degree line indicates a satisfactory model in which the predicted probabilities are identical to the actual outcomes; **(D)** Decision curve analysis demonstrating the clinical benefit gain of the nomogram for the three- and 5-years OS: the *y*-axis measures the net benefit, which is calculated by summing the benefit (true positives) and subtracting the harms (false positives). The solid line indicates the prognostic model, and the two other lines indicate the “intervention for all” (dotted line) and “intervention for none” (black line). A model is considered of clinical value if it has a higher net benefit than other models at any given threshold.

Moreover, the FerRLSig and other universal clinical characteristics were incorporated into a prognostic nomogram to better predict the one, three and 5 years’ OS probabilities (Figure 6A). Overall, the nomogram demonstrated satisfactory AUC on 1, 3, and 5 years’ OS and calibration (AUC: 1-year 0.668, 3-years 0.761, 5-years 0.779, Figures 6B,C). In addition, the nomogram also demonstrated net benefit gain in decision curve analysis compared to both “intervention to none” and “intervention to all” in both 3 and 5 years’ OS prediction (Figure 6D).

### The correlation between the FerRLSig and tumor immune microenvironment

**FIGURE 7:**
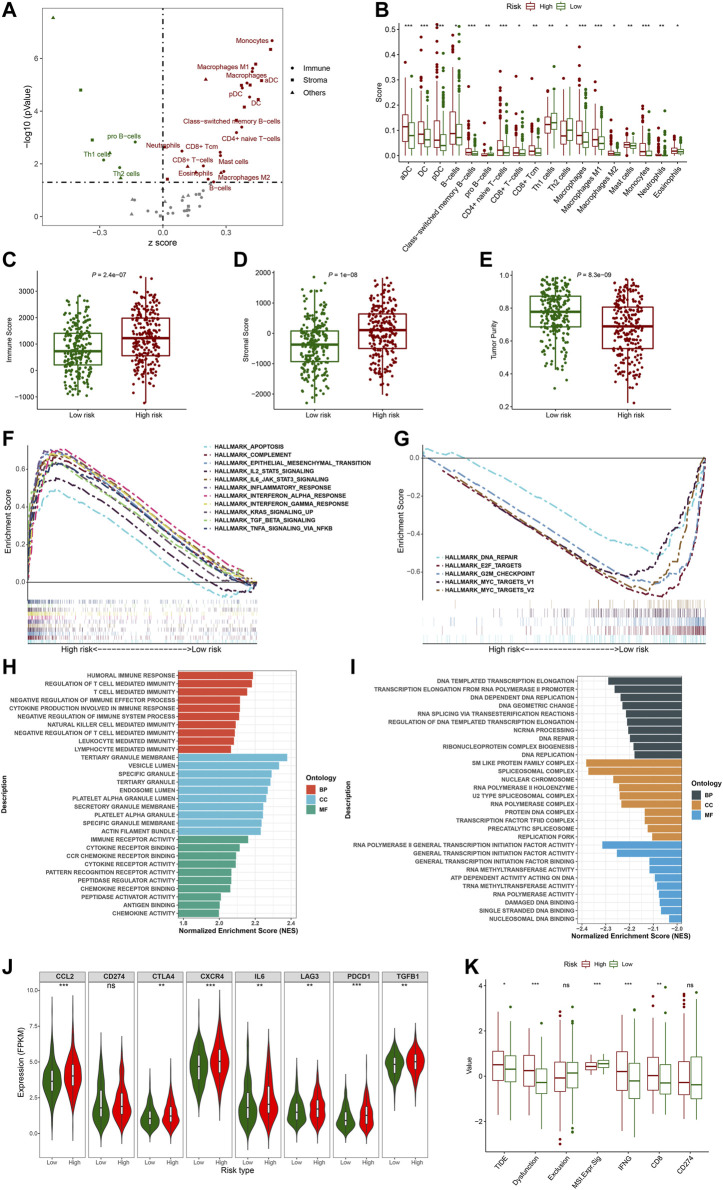
Evaluation of the tumor immune microenvironment based on the ferroptosis-related lncRNAs signature stratification; **(A)** The volcano plot depicted the different cellular landscapes of the tumor based on the ferroptosis-related lncRNAs signature (FerRLSig) stratification by the xCell analysis, the infiltration of green dot cells were significantly higher while red dot cells were significantly lower in low-risk group compared to high-risk group. **(B)** The boxplots depicted the significantly different infiltration of immune cell based on the FerRLSig stratification. **(C–E)** The boxplots compared the immune scores **(C)**, stromal scores **(D)** and tumor purity **(E)** based on the FerRLSig stratification by the ESTIMATE analysis. **(F–G)** GSEA results demonstrated the differential gene set enrichment in Hallmark with high-risk **(F)** and low-risk **(G)** group. **(H–I)** GSEA results demonstrated the differential gene set enrichment in C5 of biological process (BP), cellular component (CC), and molecular function (MF) in high-risk **(H)** and low-risk **(I)** groups based on the FerRLSig stratification. **(J)** Comparison of the immune checkpoints and immune inhibitory factors, including CCL2, CD274, CTLA4, CXCR4, IL6, LAG3, and PDCD1 and TGFB1based on the FerRLSig stratification in violin plots and boxplots. **(K)** Comparison of TIDE score based on the FerRLSig stratification in boxplots. *: *p* < 0.05, **: *p* < 0.01, ***: *p* < 0.001.

To explore the correlation between the FerRLSig and the tumor immune microenvironment, immune cell infiltration was inferred and compared between the high-risk and low-risk group with xCell analysis. The two groups exhibited apparently different tumor immune microenvironment. The high-risk group demonstrated significantly higher dendritic cells, B cells, class-switched memory B cells, CD8^+^ T cells, and multiple myeloid cells infiltration while the low-risk group had significantly higher pro B cells, Th1 cells and Th2 cells infiltration (Figures 7A,B). Moreover, both immune score and stromal score were significantly higher while tumor purity was significantly lower in high-risk group, confirming higher immune cell infiltration in high-risk group (Figures 7C–E). Furthermore, hallmark gene set enrichment analysis was performed to further characterize the different molecular pathways activated by different risk groups. Multiple immune-related hallmarks were significantly enriched in high-risk group, including complement, IL2-STAT5 signaling, IL6-JAK-STAT3 signaling, inflammatory response, interferon-alpha response, interferon-gamma response, TGF-BETA signaling and TNFA signaling *via* NFKB were enriched in high-risk group. On the other hand, low-risk group demonstrated multiple proliferation-related hallmarks and DNA damage repair hallmarks (Figures 7F, G). In addition, Gene Ontology pathway enrichment analysis also corroborated the above finding that high-risk group demonstrated multiple significantly enriched immune-related biological process and molecular function, while low-risk group was correlated with multiple proliferation and transcription ones(Figures 7H, I).

Apparently, the FerRLSig was strongly correlated with tumor immune microenvironment and high-risk group demonstrated increased immune activities compared to low-risk group. We continued to compare the expression level of immune checkpoints and immune inhibitory factors between the two groups to evaluate the potential predictive application of the FerRLSig on the current immune checkpoint blockade therapy. We found that the high-risk group was significantly associated with higher expression level of immune checkpoints and immune inhibitory factors, including CCL2, CTLA4, CXCR4, IL6, LAG3, PDCD1, and TGFB1, indicating tumor immune evasion (Figure 7J). We utilized the TIDE to further evaluate the tumor immune microenvironment of different FerRLSig groups. And surprisingly, although high-risk group consistently demonstrated higher effector T cell signatures including IFNG (interferon gamma) and CD8^+^ T cell infiltration, the T cell dysfunction signature were significantly higher while microsatellite instability score was significantly lower in high-risk group. This ultimately led to significantly higher overall TIDE score in high-risk group, indicating resistance to immune checkpoint inhibitors (Figure 7K).

### Drug sensitivity screening based on the FerRLSig

**FIGURE 8:**
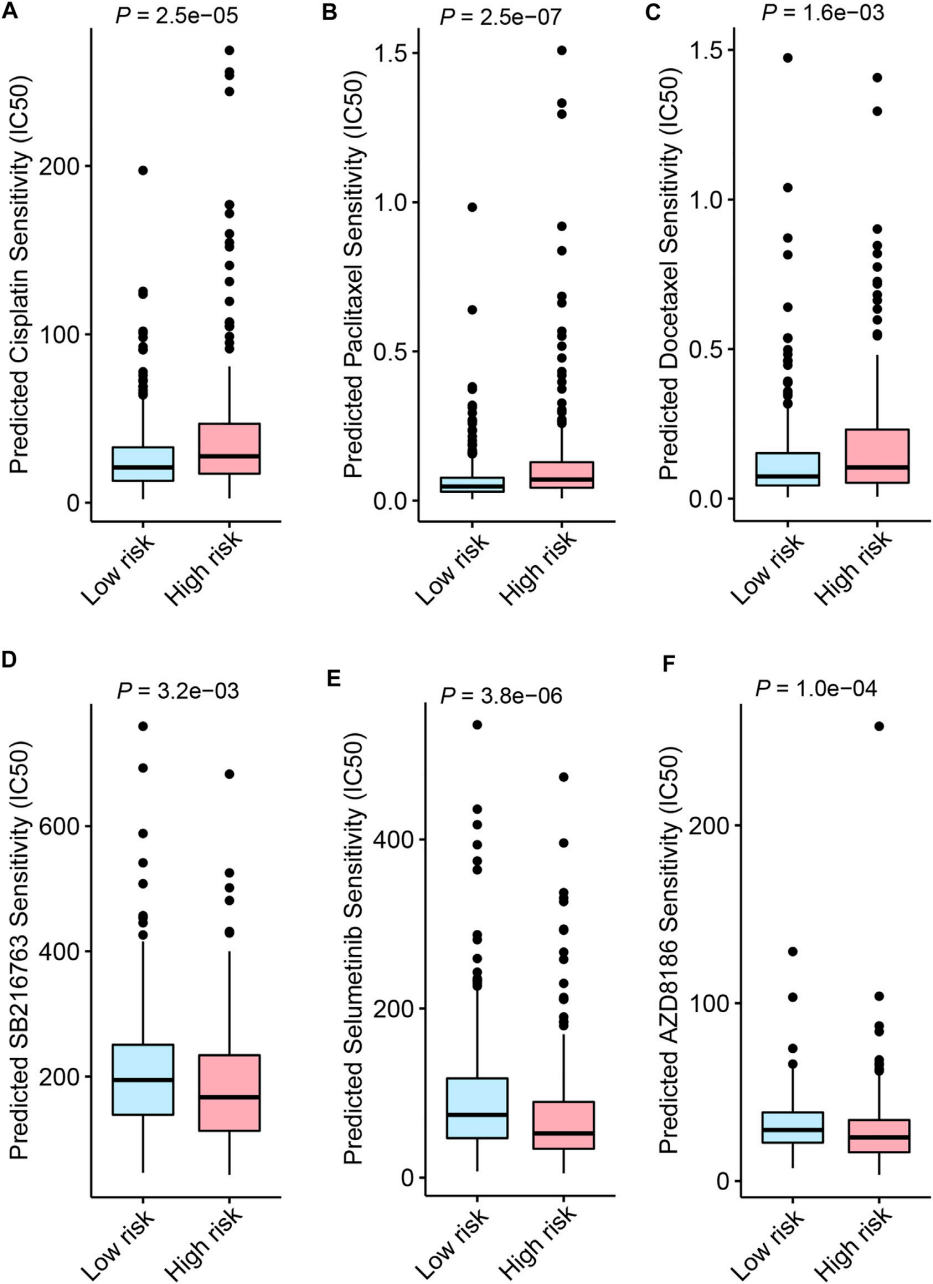
Drug sensitivity screening based on the ferroptosis-related lncRNAs signature (FerRLSig) stratification with the Genomics of Drug Sensitivity in Cancer (GDSC) database; **(A)** Boxplot of the cisplatin half-maximal inhibitory concentration (IC50) based on the FerRLSig stratification; **(B)** Boxplot of the paclitaxel IC50 based on the FerRLSig stratification; **(C)** Boxplot of the docetaxel IC50 based on the FerRLSig stratification; **(D)** Boxplot of the SB216763 (targeting WNT signaling pathway) IC50 based on the FerRLSig stratification; **(E)** Boxplot of the Selumetinib (targeting MAPK signaling pathway) IC50 based on the FerRLSig stratification; **(F)** Boxplot of the AZD8186 (targeting PI3K signaling pathway) IC50 based on the FerRLSig stratification.

Besides immune checkpoint inhibitors, we also aimed to evaluate the predictive application of the FerRLSig on other drugs available from GDSC database. Therefore, drug sensitivity screening with GDSC was performed based on the FerRLSig. We identified 119 compounds from GDSC to have statistically significant different half-maximal inhibitory concentration (IC50) based on the FerRLSig stratification (Supplementary Table S1). Notably, low-risk group was significantly more sensitive to platinum and taxane compared to high-risk group (Figures 8A–C), which are the backbones for LUSC chemotherapy, therefore might account for the superior OS of the low-risk group. On the other hand, high-risk group was seemingly more intractable with fewer clinically available systemic therapies compared to low-risk group. Three representative drugs with significantly lower IC50 in high-risk group compared to low-risk group were identified, targeting WNT signaling, MAPK signaling and PI3K signaling pathway (Figures 8D,E).

## Discussion

Previous studies have successfully developed prognostic ferroptosis-related lncRNAs signatures in lung adenocarcinoma (Guo et al., 2021; Lu et al., 2021; Zheng et al., 2021). However, the investigation of the ferroptosis-related lncRNAs signature in lung squamous cell carcinoma (LUSC) has been scare and has not evaluated the signature’s impact on tumor immune microenvironment yet. In this study, we developed and validated a ferroptosis-related lncRNAs signature (FerRLSig) for the prognosis stratification of lung squamous cell carcinoma (LUSC). High-risk group had significantly worse OS compared to low-risk group (HR = 2.240, 95%CI: 1.845–2.720, *p* < 0.001), which was further corroborated in different age, gender, ethnicities and TNM stages subgroups, indicating the wide applicability and independent prognostic effect of the FerRLSig. And notably, compared to TNM stage, the FerRLSig demonstrated consistently improved AUCs (5-years AUC: FerRLSig 0.756, TNM stage 0.607, Figures 4E,F) on OS. Thus through this study, we have developed and validated a robust prognostic ferroptosis-related lncRNAs signature for LUSC.

A previous retrospective study utilizing TCGA database identified 29 ferroptosis-related lncRNAs with univariate Cox regression and constructed a prognostic ferroptosis-related lncRNAs. The 1-, 2-, and 3-years area under curve (AUC) of their signature were 0.658, 0.693, and 0.687 respectively (Yao et al., 2022). In our study, the TCGA-LUSC patients were randomized into a training set and a testing set by the “caret” R package at the ratio of 7:3. The FerRLSig was established with the training set while the validation was performed with the testing set and the entire set. Univariate Cox regression, Least Absolute Shrinkage and Selection Operator (LASSO) regression and multivariate Cox regression were applied in order to establish the final FerRLSig while avoiding overfitting with cross-validation. A more concise ferroptosis-related lncRNAs signature comprising 10 ferroptosis-related lncRNAs with an AUC of 0.756 for 5-years OS was established and validated. Compared to the previous study, we applied more stringent statistical methods to identify ferroptosis-related lncRNAs to avoid overfitting. Moreover, we underwent additional internal validation, which was absent in the previous study. Therefore, we believed our FerRLSig to be more statistically stringent with better prognostic effect compared to the previous study.

More importantly, we have also evaluated the correlation between the FerRLSig and the tumor immune microenvironment. Generally, high-risk group demonstrated significantly higher immune cell infiltration in the xCell analysis, notably by dendritic cells, CD8^+^ T cells, M1 macrophages and M2 macrophages. On the other hand, low-risk group demonstrated significantly higher pro B cells, Th1 cells and Th2 cells infiltration. In addition, the ESTIMATE and GSEA analysis also corroborated previous results that high-risk group had significantly higher immune and tumor scores with multiple immune-related gene sets enrichment compared to low-risk group, including complement, IL2-STAT5 signaling, IL6-JAK-STAT3 signaling, interferon-alpha response, interferon-gamma response, TGF-BETA signaling and TNFA signaling *via* NFKB. CD8^+^ cytotoxic T cells and its secreted interferon gamma are central to the tumor immune elimination, and increased immune cell infiltration indicates prominent immune response, but these do not necessarily lead to better tumor control or survival (Gajewski et al., 2013; Mojic et al., 2017; Morad et al., 2021). In addition, multiple immune inhibitory factors seen in high-risk group, including M2 macrophages and IL6, might render the infiltrating CD8^+^ T cell dysfunction and leading to immune evasion (Xue et al., 2014; Kumari et al., 2016). To further evaluate the mechanism of the immune evasion based on the FerRLSig stratification, several immune checkpoints and immune inhibitory factors including CCL2, CD274 (PD-L1), CTLA4, CXCR4, IL6, LAG3, PDCD1 (PD-1), and TGFB1 were compared between low-risk and high-risk group, and all were significantly higher in high-risk group except PD-L1, strongly suggesting the immune evasion and inhibitory microenvironment in high-risk group.

Immune checkpoint inhibitors have made remarkable breakthroughs in LUSC and significantly improved the LUSC patients’ survival (Brahmer et al., 2015; Paz-Ares et al., 2018). Considering the immune evasion and inhibitory microenvironment in high risk group based on the FerRLSig stratification, TIDE score was utilized to estimate the immune checkpoint inhibitor sensitivity. CD8^+^ T cell infiltration and interferon gamma signature were significantly higher in high-risk group while no statistically significant difference was found on CD274 (PD-L1) signature, which corroborated previous results. And high-risk group was significantly less sensitive to immune checkpoint inhibitor with significantly higher TIDE score and dysfunction score compared to low-risk group. Besides the inherent limitations of the TIDE analysis, one possible explanation would likely be that the T cell dysfunction with multiple alternative immune checkpoints including CTLA-4 and LAG3 within the tumor microenvironment is beyond the salvage of the single-target immune checkpoint inhibitor (Thommen and Schumacher, 2018). Therefore, the trials of combination immunotherapy targeting multiple immune checkpoints and further innovation are needed for the future improvement of LUSC patients. Drug sensitivity screening was also performed based on the FerRLSig with drugs available from GDSC database. Low-risk group was significantly more sensitive to platinum and taxane compared to high-risk group, which might partially account for its better OS. On the other hand, high-risk group were significantly more sensitive to three representative drugs that targeting WNT signaling, MAPK signaling and PI3K signaling pathways. And intriguingly, all three pathways are known to cancer immune evasion (Sumimoto et al., 2006; Dituri et al., 2011; Martin-Orozco et al., 2019) and these kinds of drugs might be combined with immunotherapy to further improve the survival for LUSC patients. However, further investigation is needed to verify these possibilities.

Several limitations are worth mentioning in this study. Firstly, this is a retrospective study from a single database, thus external validation and further prospective study are required. Secondly, several important clinical variables including the extent of resection, resection margin, comorbidities are currently unavailable, which warrant further investigation in future studies. Thirdly, the distance to actual clinical application remains long as whole transcriptome RNA sequencing for lncRNAs identification has not been easily accessible in clinical practice yet. The last but not the least, both *in vitro* and *in vivo* experiments are required to further explore the molecular mechanism underlying the ferroptosis-related lncRNAs signature.

In conclusion, a robust prognostic FerRLSig for LUSC has been developed and validated, demonstrating a close association not only with tumor immune cell infiltration, but also with T cell dysfunction and immune evasion. Further investigation and innovation are required to validate the results from our study and better improve the survival of LUSC patients based on the FerRLSig stratification.

## Data Availability

Publicly available datasets were analyzed in this study. This data can be found here: https://portal.gdc.cancer.gov/.
