# Supplementary figures and images for: Ferroptosis-related lncRNAs signature to predict the survival and immune evasion for lung squamous cell carcinoma
Source: Front Genet. 2022 Aug 26;13:968601. doi: 10.3389/fgene.2022.968601 (PMC9459014; doi:10.3389/fgene.2022.968601)

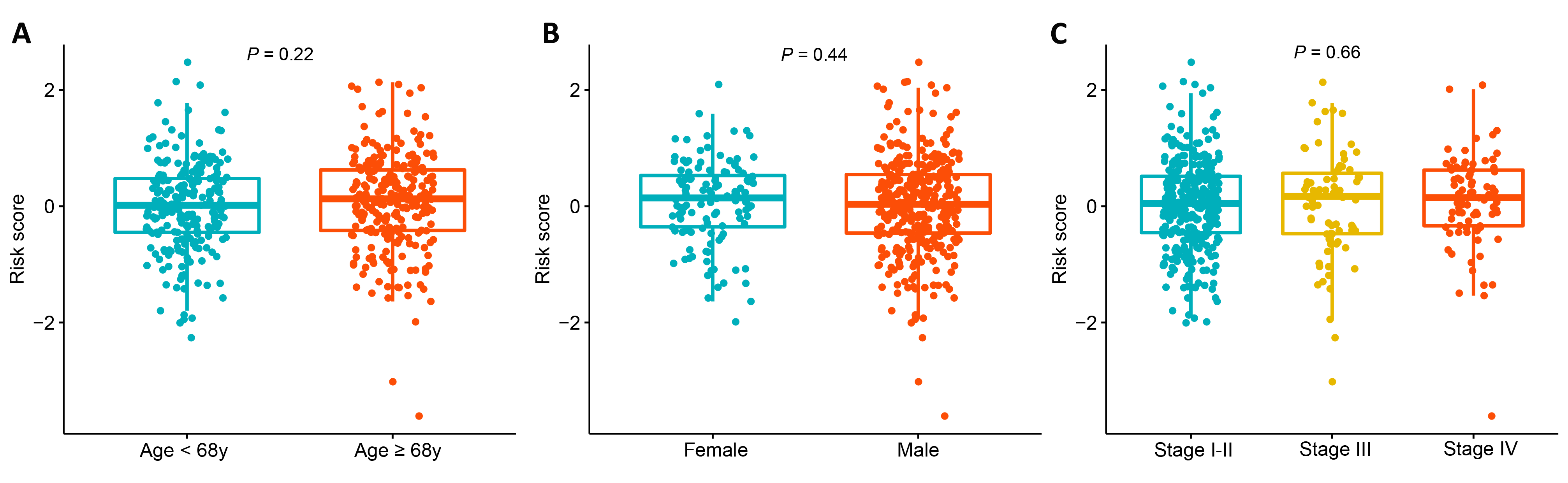

Supplement: Supplementary file 1 [file Image3.TIF]

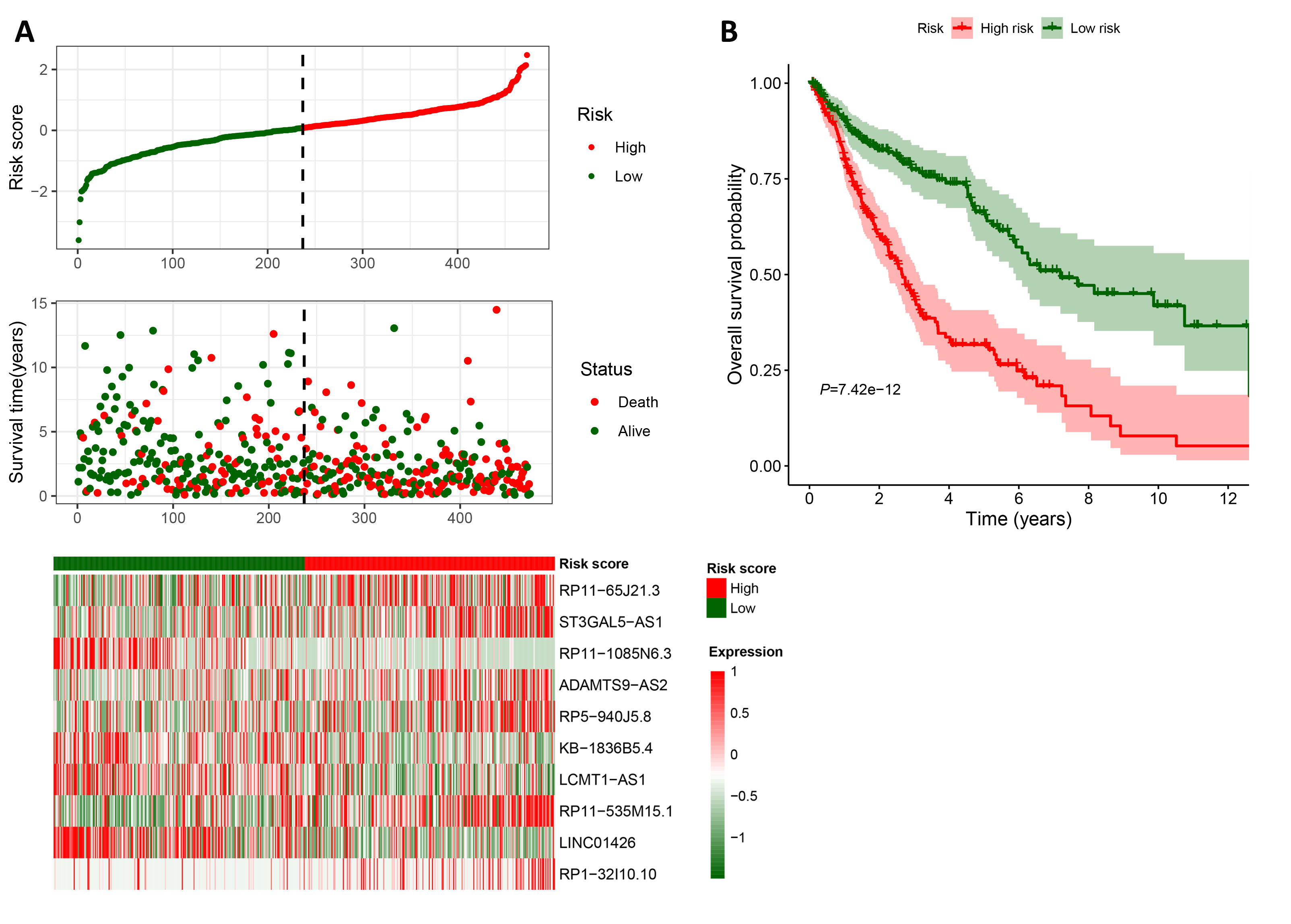

Supplement: Supplementary file 2 [file Image2.TIF]

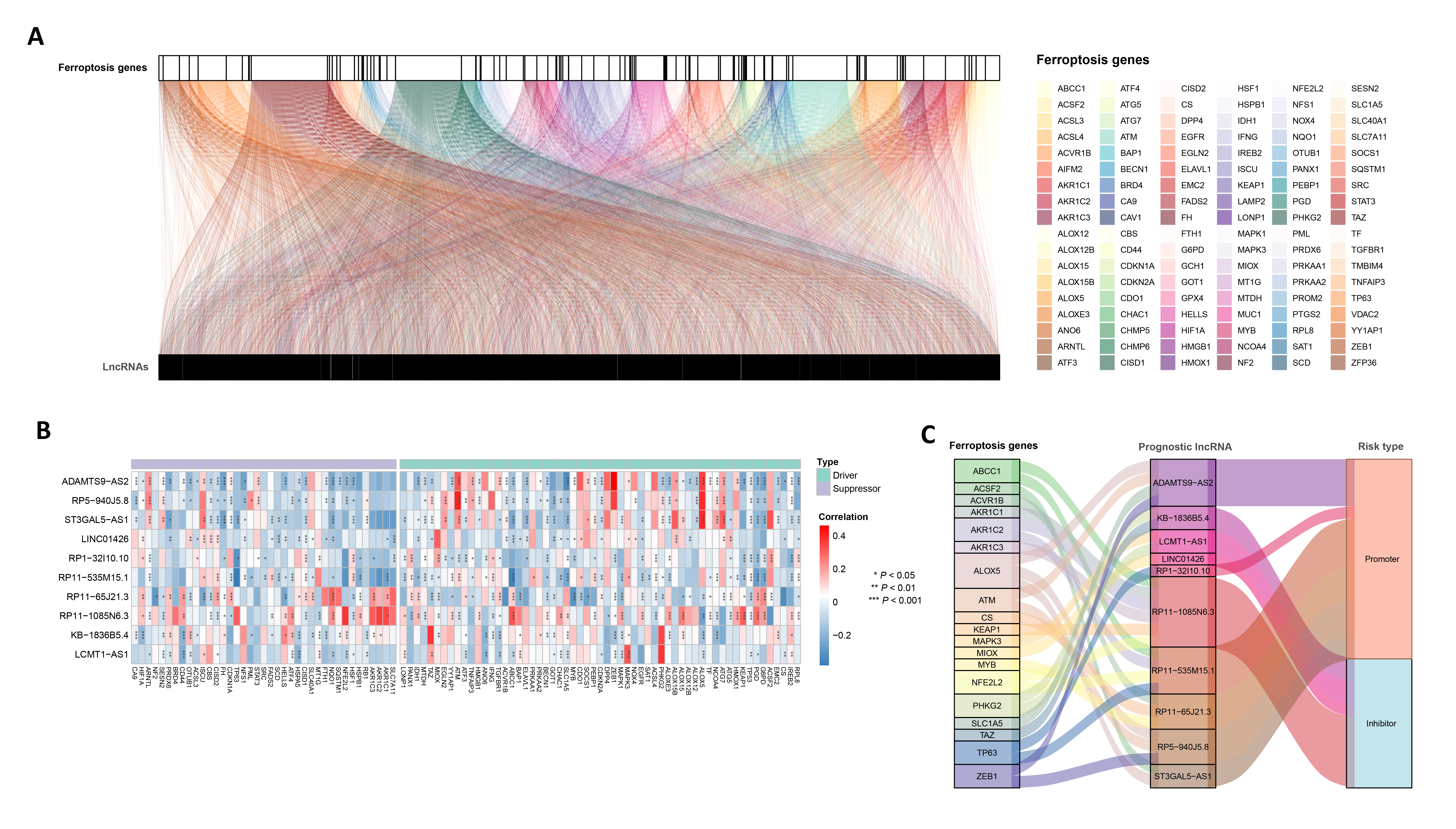

Supplement: Supplementary file 3 [file Image1.TIF]
