# Supplementary material for: Ferroptosis-related lncRNAs signature to predict the survival and immune evasion for lung squamous cell carcinoma
Source: Front Genet. 2022 Aug 26;13:968601. doi: 10.3389/fgene.2022.968601 (PMC9459014; doi:10.3389/fgene.2022.968601)
Supplement: Supplementary file 5 [file DataSheet1.docx]

Supplementary Figure 1. Visualization of the ferroptosis-related lncRNAs signature (FerRLSig) lncRNAs and the ferroptosis genes; (A) Sankey diagram for the correlation between validated ferroptosis genes from FerrDb database and ferroptosis-related lncRNAs. (B) Correlation heatmap between 10 prognostic ferroptosis-related lncRNAs and ferroptosis genes annotated with the effect of promoting (driver) or inhibiting (suppressor) ferroptosis. (C) Sankey diagram of significantly correlated ferroptosis-related genes (P < 0.001, |R| > 0.3) and 10 prognostic ferroptosis-related lncRNAs, which were classified as risk promoters or inhibitors according to their positive or negative coefficients

Supplementary Figure 2. (A) Distribution of risk score, OS time, OS status and heatmap of the 10 prognostic ferroptosis-related lncRNAs signature in the TCGA-LUSC entire set. (B) Kaplan-Meier survival curves of the OS of the patients in the high- and low- risk groups for the TCGA-LUSC entire set

Supplementary Figure 3. Correlation between ferroptosis-related lncRNAs signature risk score and age, gender and TNM stage
